# Knowledge, attitudes and practices about contraception amongst schoolgirls aged 12–14 years in two schools in King Sabata Dalindyebo Municipality, Eastern Cape

**DOI:** 10.4102/phcfm.v5i1.509

**Published:** 2013-10-15

**Authors:** Pamela Mda, Don O'Mahony, Parimalarani Yogeswaran, Graham Wright

**Affiliations:** 1 General Practitioner, Mthatha, South Africa; 2 Department of Family Medicine, Walter Sisulu University, South Africa; 3 Faculty of Health Sciences, Walter Sisulu University, South Africa

## Abstract

**Background:**

In South Africa the teenage fertility rate is high. About 42% of women have their sexual debut by 18 years of age and 5% by 15. These young women are also at risk of sexually transmitted infections (STIs) and human immunodeficiency virus (HIV) infection. Despite widespread availability of contraception, 18% of sexually active teenagers do not use any. Previous research on the knowledge of, attitudes to and practices of contraception by teenagers has focused on older adolescents.

**Objectives:**

This study explored knowledge, attitudes and practices about contraception amongst 12–14 year old unmarried schoolgirls with a view to inform planning of programmes to assist in reducing teenage pregnancies.

**Methods:**

A qualitative study design with purposive sampling was used to select participants from two government-run schools in King Sabata Dalindyebo Municipality. In-depth and focus group interviews were conducted after obtaining written consent from parents and assent from participants. Interviews were audiotaped, transcribed verbatim, translated and analysed thematically.

**Findings:**

Participants reported that young adolescents were sexually active, which included high-risk sexual behaviour such as multiple partners and casual and transactional sex. Knowledge about contraceptives varied widely. Condoms were the most preferred method of contraception, but it is unknown whether they ever used condoms as they professed to talk about the behaviour of others rather than themselves. Injectable contraceptives were believed to have long-term negative effects. Common sources of contraceptive information were friends or peers, school curriculum and to a lesser extent family members.

**Conclusions:**

Findings of the study suggest that young adolescents are sexually active and have inadequate knowledge and misconceptions about contraception. These findings should inform educational programmes about risks of early sexual activity and about contraception.

## Abstrait

**Connaissances, attitudes et pratiques relatives à la contraception chez les collégiennes âgées de 12 à 14 ans dans deux écoles de la municipalité de King Sabata Dalindyebo, Cap oriental**

**Contexte:**

Le taux de fécondité chez les adolescentes sud-africaines est élevé. Environ 42% des femmes commencent à être sexuellement actives à 18 ans et 5% à 15 ans. Ces jeunes femmes courent également le risque de contracter des infections sexuellement transmissibles (IST) et le virus de l'immunodéficience acquise (VIH). En dépit de la disponibilité largement répandue des modes de contraception, 18% des adolescentes sexuellement actives n'en utilisent aucun. Les études antérieures réalisées sur les connaissances, les attitudes et les pratiques relatives à la contraception chez les adolescentes se sont concentrées sur les plus âgées d'entre elles.

**Objectifs:**

Cette étude s'est intéressée aux connaissances, attitudes et pratiques relatives à la contraception chez les collégiennes âgées de 12 à 14 ans et non mariées afin d'informer la planification des programmes destinés à réduire les grossesses chez les adolescentes.

**Méthodes:**

Une méthode d’étude qualitative, avec échantillonnage dirigé, a été utilisée afin de sélectionner les participantes issues de deux écoles publiques dans la municipalité de King Sabata Dalindyebo. Des entretiens approfondis ont été réalisés et des groupes de discussion organisés après avoir obtenu le consentement des parents et l'assentiment des participantes. Les entretiens ont été enregistrés au format audio, retranscrits mot à mot, traduits et analysés selon des thématiques.

**Résultats:**

D'après les participantes à l’étude, les jeunes adolescentes étaient sexuellement actives et adoptaient des comportements sexuels à haut risque comme avoir des partenaires multiples et des rapports sexuels occasionnels ou transactionnels. Les connaissances sur les méthodes de contraception variaient dans une large mesure. Les préservatifs constituaient la méthode de contraception privilégiée, mais on ne sait pas si elles les utilisaient, puisqu'elles parlaient du comportement des autres plutôt que du leur. Les méthodes de contraception injectables étaient considérées comme ayant des effets négatifs sur le long terme. Les sources d'informations généralement disponibles sur les méthodes de contraception étaient généralement les amies ou les paires, le programme scolaire et, dans une moindre mesure, les membres de la famille.

**Conclusions:**

Les conclusions de l’étude suggèrent que les jeunes adolescentes étaient sexuellement actives et disposaient de connaissances inadéquates et d'idées fausses sur la contraception. Ces conclusions devraient informer les programmes éducatifs sur les risques d'une activité sexuelle précoce et la contraception.

## Introduction

### Key focus

Worldwide the rate of teenage pregnancy has decreased. In South Africa the teenage fertility rate was estimated as 66 out of 1000 women in 2007^1^ and 54 out of 1000 in 2010.^2^ However, the rate is still high compared to more developed countries; for example, in the United States of America (USA) the rate is 39.1 out of 1000 in 15–19 year olds.^3^ The USA has the highest rate amongst the industrialised countries; the rate in the United Kingdom was 30 out of 1000 and in Germany 7 out of 1000 women in 2010.^2^

Teenage pregnancy is associated with discontinuation of schooling and reduced prospects of employment.^1, 4^ In addition, early reproduction is associated with increased risk of contracting human immunodeficiency virus (HIV) infection in the South African context.^1^ Worldwide, pregnancy-related complications including childbirth complications and unsafe abortions are leading causes of death in the age group 15–19 years. Girls younger than 15 have been reported to be at five times higher risk than their counterparts aged 20 years and above.^5^

### Background

Adolescence as defined by the World Health Organization (WHO) is the period between 10–19 years of age, further subdivided into early (10–14 years) and late (15–19 years) adolescence.^6^ This period is characterised by a rapid rate of physical growth associated with development of secondary sexual characteristics as a result of hormonal influence.^7^ Adolescents account for 20% of the global population, with the majority in developing countries.^6^ The South African population was estimated at 50.6 million in 2011, of whom 10.5 million are adolescents.^8^

Although adolescents have low disease incidence and mortality, exploration, opportunity and risk-taking behaviour are common characteristics of this group.^9^ Negative consequences of their risk-taking behaviour include unintended pregnancies, abortion and sexually transmitted infections (STIs) including HIV and acquired immune deficiency syndrome (AIDS), substance use and abuse and injuries.^10, 11^ Whilst there are variations amongst countries, one-third or more unmarried adolescent girls in developed countries and in sub-Saharan Africa (except Rwanda and Nigeria) have had sexual intercourse.^11^

In South Africa the second South African National Youth Risk Behaviour Survey (SANYRBS), undertaken in 2008^9^ amongst secondary school learners, found that 38% had had sex. For 13% of the adolescents age of sexual initiation was before14 years.^9^ The 2003 South African Demographic and Health Survey reported that 42% of women had had their first sexual encounter before the age of 18 years.^12^ Additionally, 21% of young men and 11% of young women in the Eastern Cape had first sexual intercourse by the age of 15 years^12^ – the lowest age of sexual debut in all nine provinces.

According to the 2008 SANYRBS only 45% of the sexually active teenagers used a condom for contraception during the last sexual intercourse, whilst 18% of learners did not use any form of contraception. This shows a marked improvement from the 2002 SANYRBS, which revealed 28% condom use.13 Prevalences of other contraceptive methods used by learners were as follows: injectable contraceptive 7.0%, birth control pill 4.7%, withdrawal 3.3%, other methods 2.6%, and emergency contraceptive 1.4%.^9^ Of learners in the 14 year old group, 44.8% did not use any form of contraception compared to 25.1% in the 19 year old group.^13^

During early adolescence young boys and girls undergo cognitive, emotional, sexual and psychological transformation in that they become aware of their gender. This may result in change of behaviour and appearance to fit the social norms of their peers.^6^ This is a critical stage for targeting young adolescents for primary prevention of unintended pregnancy, STIs and HIV infection and AIDS.^14^

The WHO acknowledges that the sexuality of adolescents between the ages of 15 and 19 years has been extensively researched. However, younger adolescents’ sexuality has rarely been explored, despite knowledge that sexual activity has begun.^15^

### Trends

Many intervention programmes have tried to encourage behaviour change in young people to prevent unintended pregnancies and HIV infection in sub-Saharan Africa, with school-based education on HIV and AIDS, STIs and general reproductive health issues.^16^ However, primary prevention strategies and interventions have been disappointing, and did not change adolescent behaviour in respect of delaying initiation of sexual intercourse, uptake of contraception and rate of teenage pregnancies.^17^

In 1999 the South African National Department of Education launched a programme aimed at addressing educational, health and social needs of learners. The programme included sexuality, gender, school safety, health and skills development.^9^ The end result was introduction of Life Orientation (LO) as a compulsory learning area from Grades 1 to 12.^18, 19^ LO comprises a wide range of components: guidance, life skills education including HIV and AIDS education, health promotion, physical development and movement, environmental education, citizenship and human rights and religious education.^18^

## Objectives

This study aimed at exploring knowledge, attitudes and practices about contraception amongst 12–14 year old unmarried schoolgirls with a view to informing planning of programmes to assist in reducing teenage pregnancies.

## Contribution to the field

The findings identified a need for further research into specific aspects of contraception in this age group. Policy makers may use the findings to inform age-appropriate interventions in current or future programmes.

## Research method and design

### Subjects

In-depth semi-structured and focus group interviews were conducted to collect data. An interview guide was utilised to gather information during individual interviews; this was designed and informed following a literature review.^20^ The interview guide covered questions on: (1) understanding of sexuality; (2) knowledge about STIs and HIV and AIDS; (3) knowledge about teenage pregnancy; (4) knowledge, attitudes and practices about contraception; and (5) the role of the mass media on sexual behaviour.

### Setting

The study was conducted in King Sabata Dalindyebo Local Municipality (KSD), the biggest local municipality in Oliver Reginald Tambo District Municipality in the Eastern Cape Province, South Africa. King Sabata Dalindyebo Local Municipality encompasses two towns, namely Mthatha and Mqanduli. One government-run (co-educational) school from each town was selected.

### Design

A qualitative study design was used

### Procedure

Life Orientation teachers recruited schoolgirls in the 12–14 year age group at each school using purposive sampling. This allowed selection of appropriate participants who represented a range of beliefs and experiences relevant to the research topic. In-depth semi-structured interviews were conducted in isiXhosa, the participants’ language of choice. The researcher asked open-ended questions, giving the participants freedom to express their views in their own words. Interviews were stopped after data saturation was reached.

Two focus group interviews (one in each school) were conducted, each with seven participants. The researcher facilitated the interviews whilst a research assistant took field notes and audiotaped proceedings.

### Analyses

The research assistant transcribed the audiotapes verbatim and translated them into English. Transcripts were read and re-read as the researcher familiarised herself with the content of the interviews. Line numbers were used to identify questions asked by the interviewer and the participant's response. Themes were developed from participants’ responses, informed by the broader study objectives. Responses were categorised according to themes and content analysis was used to interpret them.

## Ethical considerations

Ethical approval was obtained from the Walter Sisulu University Health Research Ethics and Bio-safety Committee (clearance certificate 045/011). The school principals and governing bodies granted permission to conduct the study in each school.

### Potential benefits and hazards

A numbering system used during data collection and analysis ensured confidentiality and anonymity.

### Recruitment procedures

Consenting parents or guardians counter-signed assent forms completed by willing participants prior to the study. No incentives were given to participants or school staff.

### Informed consent

Parents or legal guardians were provided with written information about the study and details of their children's participation. Rights of parents to refuse participation by their children were highlighted. Participants were also provided with an information sheet, which highlighted the objectives of the study and their right to withdraw from the study at any stage without negative consequences.

### Data protection

Access to data was limited to the researcher and research assistant. Transcripts were kept secure in a locked cabinet in the researcher's office.

## Trustworthiness

### Reliability and validity

The validity of the study was ensured by triangulation: use of in-depth interviews, focus group interviews and field notes. An independent observer fluent in English and isiXhosa listened to random portions of the audiotaped interviews and compared transcribed and translated data for accuracy and completeness, which were confirmed. The results cannot be generalised across the KSD as this was a qualitative study, but results may be transferrable to similar settings

## Results

There were 25 participants: 8 were 12 years old, 12 were 13 years old and 5 were 14 years old. Participants were in Grades 7 and 8 at the time of data collection in December 2011. Data saturation was reached after the eleventh in-depth interview, and important themes were identified.

It should be noted that in both in-depth and focus group interviews participants stated their reluctance to talk about their own sexual behaviour, but volunteered to talk about family or friends of their own age.

### Sexual behaviour

Participants described types of relationships they knew, which included dating with or without sexual relations, multiple sexual relationships, casual sex and prostitution. Eleven participants knew at least one teenage girl of their age who was dating and engaged in sexual intercourse. It was apparent that teenage girls had preferences concerning the age of their boyfriends; some dated schoolboys whilst others dated older working men. As two participants stated:

‘All of us in this group know someone who is dating and having sex, but others are shy because they are friends with them.’ (PIM, Grade 7, age 13)

‘My neighbour is 14 this year, she sleeps with boys, not just one boy.’ (P3U, Grade 7, age 13)

Prostitution was mentioned in focus groups, most agreeing that it was a fairly common practice amongst teenagers:

‘Some prostitutes [*young girls*] want to provide money and food for their siblings [*child- headed homes*]; in some cases some prostitutes just want to have money.’ (P9U, Grade 8, age 13)

### Reasons for sexual engagement

When asked what motivated their peers to start dating and become sexually active, responses were fast and spontaneous in both in-depth and focus group interviews. Most commonly mentioned was access to items such as money, airtime, gifts and clothes:

‘Girls date old men because they want money and also these guys have cars. They would be given airtime when they want it.’ (P1U, Grade 7, age 12)

Some mentioned that boyfriends put pressure on girls to become sexually active. Some boys would threaten to leave the girl if she did not engage in sexual activity:

‘The girls who are dating older men are sometimes forced to do this thing... sex.’ (P1U, Grade 7, Age 12)

Other girls apparently also put their friends under pressure to have sex by either isolating them or calling them demeaning names.

The mass media was also reported to play a role in teenagers’ sexual behaviour. The most common types known by participants were television, radio and magazines, and of these television was cited as most influential on sexual behaviour. All participants had television sets in their households; few had radios and they rarely listened to them.

Programmes specifically designed for their age group were found to be less interesting as they took the form of precautionary and educational warnings. Most participants reported that these were boring and some would only watch them in the presence of an adult. All understood age restrictions on television programmes, but most said they ignored them:

‘I just disregard the age restriction, as long as there is information in that thing, in fact if that programme has things like kissing that's exactly what we would love to watch.’(P13U, Grade 8, age 13)

‘Another thing that makes teenagers reluctant to watch these [*age-restricted programmes*] is their experience, some of these programmes teach them about abstaining from sex when they know that sex is very nice.’(P7U, Grade 8, age 13)

Some participants mentioned magazines as a good source of information about dating, with tips on choosing a date and question and answer sections on relationships and sexual matters, whilst pictures influenced the reader to experiment.

Mobile phones as an electronic form of media proved to be easily accessible, as 21 of the participants owned one. Most used mobile phones for basic communication, but some were aware that they could be used to distribute and share pornographic material.

### Communication with parents about sex and relationships

Most participants agreed that parents were not happy with young girls being involved in relationships. Some mentioned that their mothers shouted at them whenever they talked about sexual matters and they did not like that:

‘I think the parents should talk to us in a nice way and not shout at us because if they shout at us we will have some fears regarding sharing some things with them …’ (P9U, Grade 8, age 13)

‘It would be better if your parent would sit down with you and talk to you instead of shouting at you about hearsays.’ (P7U, Grade 8, age 13)

### Attitudes towards teenage pregnancy

Most participants were aware that teenagers do fall pregnant, and some knew teenagers their age (12–14 years) who had been or were currently pregnant. Reported reasons for pregnancy at this age ranged from ignorance about contraception to informed choice, coercion, rape and prostitution:

‘There is this girl in my neighbourhood, she had her first child when she was 14 years old. She is now 16 years old and pregnant with her second baby.’ (P11U, Grade 8, age 14)

#### Negative effects of teenage pregnancy

Most thought the main negative effect of teenage pregnancy was disruption of schooling with long-term consequences such as shattered dreams. One acknowledged that the child becomes the girl's burden and not that of the boy who impregnated her. Another mentioned the medical implications of teenage pregnancy:

‘I think teenage pregnancy is wrong because even the boy who has impregnated you won't want to keep the child because he doesn't want to see himself burdened. He will want to proceed with his studies when you are remaining behind because of looking after the child.’ (P10U, Grade 8, age 13)

#### Child Support Grant

Participants reported that some teenagers were motivated to fall pregnant by the Child Support Grant, but others did not agree. In general participants felt that the monetary value of the Child Support Grant was too small to motivate them to fall pregnant

### Knowledge about contraception

Knowledge about contraception, commonly known as prevention (of pregnancy), was relatively limited. In focus group interviews Grade 8 learners dominated and Grade 7 contributed little. They reported that contraception was covered in the Grade 8 curriculum in Natural Science. Some received information about contraception at home (from parents, relatives, sisters & cousins), at clinics and from friends.

Participants shared their knowledge about different types of contraceptives. On average, half knew at least one type of contraceptive, including injectable contraceptives, oral contraceptives, condoms, diaphragms and intra-uterine contraceptive devices:

‘In LO we were taught about good and bad ways of preventing pregnancy, types of contraceptives that are ideal for older people and us. We were also taught about the different types such as Femidom, injection and pills. Femidom is for adults only.’ (P13U, Grade 8, age 13)

Some participants did not associate condoms with contraception – only with HIV prevention:

‘If you want to protect yourself from HIV, use a condom and if you want to avoid pregnancy, practice contraception.’ (P2M, Grade 7, age 14)

None mentioned traditional methods, sterilisation or emergency contraception, even after prompts. A few mentioned abstinence as the method of choice to prevent pregnancy.

#### Dual protection

Dual protection was an unfamiliar term to most participants. One participant who is a peer educator mentioned dual protection in a focus group interview, and as she explained what it meant her peers looked puzzled:

‘You can use contraceptives with the condom at the same time, you do it for in case the condom bursts and you will be safe from falling pregnant.’ (P13U, Grade 8, age 13)

### Attitudes about contraception

Most participants freely shared their perceptions about contraception. Most were against use of contraceptives for they believed long-term effects were undesirable, whilst acknowledging their benefit of preventing pregnancy. Some were negative about it for no reason. The most common side-effect mentioned was inability to bear children later in life, which they felt very strongly about:

‘I think it is right and wrong at the same time. It is right because you are still young so it prevents you from getting pregnant at this age. [*Raising her voice*] It is wrong because it might affect you in the future and end up not having children when you want them.’ (P11U, Grade 8, age 14)

‘People can see you when you are using contraceptives because your body muscles become shaky and wobbly. When you get married, you will not have children.’(P13U, Grade 8, age 13)

### Practices about contraception

Most teenagers did not use contraception for different reasons: some did not know about it, some knew but did not practice it. Teenagers were using different types of contraceptives, and some recommended abstinence:

‘I think the right way of preventing pregnancy is just to stop dating.’ (P9U, Grade 8, age 13)

### Accessibility of contraceptives

Most participants mentioned that condoms were the most accessible form of contraception, freely available at clinics, hospitals and public telephone booths, and they did not have to ask for permission to get them. Condoms could also be bought from shops and pharmacies. With other types of contraceptives, for example pills and the injectables, they had to speak to someone like a nurse or a doctor, which some participants viewed as a deterrent to accessing contraception. Another type of contraceptive referred to was the female injectable:

‘The thing is, contraceptive injections are given at the clinic, so what might prevent you going there is the thought that your mother's friend works in that clinic and you end up deciding not going there. In addition, adults who are attending the clinic ask you, you are this young and you want to prevent pregnancy?’ (P4M, Grade 8, age14)

‘The ones that are scarce are female condoms, the Femidom, hence people would go for male condoms.’ (P13U, Grade 8, age 13)

## Discussion

Societies differ in their approach to adolescent sexuality. In the USA adolescent sexuality is viewed as biologically driven but inappropriate and disruptive to the adolescent and family.^14, 21^ This is based on the assumption that adolescents are immature and would not associate actions with consequences, for example using contraceptives when sexually active.^14^ On the other hand, Dutch society views it as a normal phenomenon that is about love relationships and social responsibility.^14^ Within Dutch society adolescent sexual behaviour is guided by conditions such as being in a stable love relationship and using contraceptives prior to engagement in sexual activity.^14^ Dutch parents talk to their children openly about sex and casual sexual relationships are discouraged.

It is interesting to note that the USA has the highest prevalence of teenage pregnancy in the developed world,^22^ whilst The Netherlands is amongst those with the lowest prevalence.^14, 23^ South Africa and the USA have a similar approach to adolescent sexuality, and both have high pregnancy rates. The participants in this study reported that their parents did not want to discuss sexual matters and disapproved of adolescent sexual relationships.

Traditional African culture had a system of nurturing adolescent sexuality, with older girls responsible for teaching young girls once they reach puberty.^24^ Inspection of young girls’ virginity was a well accepted practice. In her ethnographic research near Lusikisiki in the 1930s, Hunter^25^ observed a young age of sexual debut (girls as young as 8 years, boys of 9 years) and open sexual relationships with the full knowledge of the parents. In fact, parents wanted to monitor the relationships to ensure that unmarried girls remained virgins by use of non-penetrative sex, and to vet potential husbands.^25^ These customary practices have now fallen away.

The level of maturity of adolescents aged 10–12 years allows them to explore their sexual curiosity with their peers, usually without personal consequences.^15^ Typically non-coital dating comprises chatting, hand-holding, kissing and petting (1% and 5%), but rarely intercourse.^26^ In this study participants who admitted dating, mentioned the non-coital relations such as chatting over the phone, sending each other text messages and holding hands with their boyfriends.

Almost all participants knew someone of their age who was engaging in sexual activity. This is consistent with a previous study that reported 11% of Eastern Cape adolescent girls had their sexual debut before the age of 15.^12^ The results suggest that the participants engage in risky sexual behaviour, as supported by their reporting on the types of sexual relationships they were involved in. Participants mentioned that sexual activity was common in their age group; some peers engaged in multiple sexual relations, and some had casual sex and transactional sex with older men. These behaviours put them at risk of teenage pregnancy and acquisition of STIs including HIV infection.

One participant used the term ‘sugar daddy’ to refer to older men who offered money and gifts in exchange for sex, a concept not limited to South Africa.^26^ It may be associated with sexual coercion as the male partner is much older than the girl. Teenagers are reported to have difficulty refusing sexual involvement with these men, who offer them gifts and money.^26^ In this study some participants also reported that girls sometimes have sex against their will.

All participants agreed that the best time to become pregnant was after completion of studies, when married. However, they did not discourage sexual activity at their young age. Despite awareness of the risks and consequences of pregnancy, they were reluctant to use contraceptives. This could indicate a willingness to take risks and or immature reasoning.

Participants in Grade 7 had less knowledge regarding contraception; this is because teaching about contraception only commences in Grade 8. Younger participants were further disadvantaged by the fact that sexually active peers did not want to share information on contraception with them. As noted by Craig and Richter-Strydom,^27^ friends are an important source of knowledge about contraception in the Black African adolescent group.

Some participants reported that due to social isolation and name calling associated with not dating, adolescents succumbed to peer pressure, and this is consistent with other South African studies.^28, 29^ Peer pressure may have positive aspects: Wood, Maepa and Jewkes^29^ report that some girls were pressured by friends to use contraceptives to avoid pregnancy.

The most commonly known methods were injectable and oral contraceptives, condoms (male and female), followed by diaphragms and loops. These are commonly available at government facilities in the Eastern Cape. Very few participants mentioned abstinence as a means of preventing pregnancy.

Most participants expressed a very strong attitude against injectable contraceptives based on their perceived side-effects, the most feared being infertility at a later stage, a finding noted in previous studies.^30, 31^ Most imagined themselves getting married and starting families and would not risk this by using an injectable contraceptive.

Participants also mentioned changes in body appearance such as ‘wobbly’ and shaking muscles as evidence of using an injection, and changes have been reported by other teenagers.^32^ Some participants did not have any reason for disliking the injectable contraceptive, but still did so.

Almost all participants knew that condoms prevent transmission of HIV infection, although very few linked condoms to pregnancy prevention, reflecting similar findings by Wood et al.^29^ Participants expressed their preference for condoms over other contraceptive methods based on accessibility, availability and wider choice. Choices included different types of condoms such as flavoured and plain, which seemed to be an attractive feature. Williamson and colleagues^31^ also noted the appealing features of condoms compared to other methods of contraception.

Participants did not mention any negative associations with use of condoms. Other studies have mentioned possible barriers to use of condoms such as fear of them being left in the vagina and the need for health professional to remove them.^33^ There is also a perceived association between use of condoms and promiscuity or presence of disease that discourages teenagers from using them.^34^

Participants in this study did not know of traditional methods of preventing pregnancy. Some studies suggest that some adolescents opt for methods such as withdrawal, periodic abstinence and use of herbal mixtures prepared by traditional healers.^29, 31^ Certain churches such as the Zion Christian Church prohibit use of contraception (biomedical) and offer an alternative in the form of tea.^29^

Various studies have reported inconsistent use and discontinuation of contraceptives by adolescents.^33–35^ Issues affecting this may be related to accessibility, availability and side-effects of contraceptives in addition to adolescents’ knowledge and attitudes. These studies also revealed that adolescents were discouraged from attending clinics for contraceptive purposes because nursing staff would shout at them. Health care workers sometimes threatened to tell the adolescents’ parents about their requests. The findings are consistent with other South African studies^32, 33, 34^ and have been acknowledged by the Department of Health^36^ as challenges of the health sector. Hartell^37^ suggests that condoms should be freely available in schools and on university campuses.

The mass media has been found to be a major source of health-related information for adolescents in many countries.^38, 39^ The media includes television, magazines and movies as well as the Internet, social networks, mobile phones and video games.^40^

Most participants mentioned that of all media, television had the most dominant influence regarding sexual behaviour. Soap operas and dramas were the most watched, followed by comedies and horror movies. This is consistent with findings in another South African study where teenagers enjoyed soap operas and television dramas.^41^ Studies report that adolescents who watch television programmes that contain sexual talk and scenes that did not have negative consequences have been found to engage in coital activities earlier than their peers.^39, 42^ Some participants in the current study expressed the opinion that television heightened their interest in experimenting.

Participants were fully aware that television programmes have age restrictions; it was apparent that an age restriction was an attraction. Despite the age restriction, some participants watched late-night programmes that show explicit sexual scenes, and some would lower the volume to prevent their parents from hearing.

They mentioned that most parents were not aware of such programmes because they retired early. Collins et al.^42^ suggest that parents should watch television with their children and discuss sexual content for the appropriate age. This study showed that participants knew age- appropriate programmes and watched them with the family, but also watched adult programmes on their own. It has been shown that even during family viewing times programmes and commercials have a significant amount of sexual content.^39^

Television is not always associated with negative sexual messages. Intervention programmes have included television as a medium of choice because of its wide coverage. Edutainment programmes such as Soul City are designed to influence viewers regarding health-related issues and increase awareness. Participants did not find Soul City programmes particularly interesting, even though presented in a melodramatic manner. Some claimed that educational programmes were boring and always relayed precautionary messages. Collins and colleagues^43^ agree that adolescents sometimes become resistant to advocacy messages. This current study suggests that it is time to re-evaluate television programmes for adolescents.

Interventions may be ineffective because parents, teachers and health care professionals are embarrassed to address issues of childhood sexuality or underestimate the value of information given in promoting healthy sexuality in youth.^44^ Life Orientation has had minimum impact on sexual behaviour of learners.^18^

## Limitations of the study

Participants preferred to share information about their friends, relatives or peers and not about themselves; therefore information bias cannot be excluded. It is not known whether the participants would provide the same information if interviewed in a different environment at a different time.

## Recommendations

Standardisation of LO at schools may provide learners with similar knowledge in a particular grade. Particular attention should be paid to correcting learners’ misconceptions about contraceptives. Lessons about contraception should be introduced early in the school curriculum, as the study suggests that adolescents become sexually active before they are equipped with information and skills. Whilst there may be concerns that such lessons may lead to early sexual experimentation, studies show that sex education programmes do not hasten initiation of sexual intercourse.^45, 46^

## Conclusion

Findings of this study suggest that young adolescent girls are sexually active. Their sexual behaviour is unsafe, as supported by the nature of the sexual relationships they reported. Inadequate knowledge about contraception and misconceptions about certain contraceptive methods were evident.

Future research should address means of improving knowledge about contraception and influencing behaviour of young adolescents before they become sexually active.
